# A novel mutation in the ubiquinol-cytochrome c reductase synthesis-like gene associated with complex III deficiency and Björnstad syndrome: A case report

**DOI:** 10.1097/MD.0000000000023026

**Published:** 2020-10-30

**Authors:** Xuncan Liu, Yanfeng Zhang, Jianmin Liang, Si Yang, Chen Chen

**Affiliations:** a Department of Rehabilitation Medicine; b Department of Pediatric Neurology, The First Hospital of Jilin University, Changchun, Jilin, China.

**Keywords:** ubiquinol-cytochrome c reductase synthesis-like, complex III deficiency, Björnstad syndrome, psychomotor developmental retardation, infantile spasms

## Abstract

**Rationale:**

The ubiquinol-cytochrome c reductase synthesis-like (BCS1L) gene is located on chromosome 2 (2q35) and encodes an ATPase that is associated with various cellular activities and is embedded in the mitochondrial inner membrane; this ATPase is presumed to facilitate the insertion of the Rieske Fe/S protein into precursors of Complex III (CIII) during the assembly of the respiratory chain. We report the first case of a compound heterozygous mutation in the BCS1L gene in China.

**Patient concerns:**

A 7-month-old girl presented with a 3-month history of psychomotor developmental retardation and a 1-month history of epilepsy combined with parallel psychomotor developmental deterioration. The clinical manifestations in the patient included psychomotor developmental retardation, infantile spasms, pili torti, tubulopathy, hepatic pathologies and lactic acidosis.

**Diagnosis:**

Combined with her clinical presentation, the patient was diagnosed with CIII deficiency and Björnstad syndrome caused by a novel mutation in the BCS1L gene after molecular biological examination. Whole exome sequencing revealed a compound heterozygous mutation with a missense mutation (c.548G > A/p. R183H) inherited from her mother and an insertion mutation (c.1061_1062insCTA/p. G354delinsGY) inherited from her father.

**Interventions:**

Before admission, the patient had received oral topiramate for 1 month. After admission, additional intravenous arginine hydrochloride was administered for five days in the acute metabolic disorder phase, and persistent cocktail therapy was introduced, including coenzyme Q10 (20 mg/d), carnitine (1 g/d) and vitamins (vitamin B1, vitamin B2, vitamin B6, and vitamin C).

**Outcomes:**

The spasm seizures were decreased by 50% after 2 weeks of treatment. The blood ammonia, myocardial enzyme and urine glucose levels declined to normal levels. At a 1-month follow-up, the patient improved clinically with a decrease in spasm seizures of 75%, stronger sucking and more voluntary activities. However, she still had mild lactic acidosis and mild hepatic damage.

**Lessons:**

We reported the first patient with CIII deficiency and Björnstad syndrome in China and identified 1 novel mutation (C.1061_1062insCTA and P. G354delinsGY) in the BCS1L gene. This finding expands the BCS1L gene mutation profile and will be beneficial for genetic diagnosis.

## 1 Introduction

Complex III (CIII), also known as the ubiquinol-cytochrome-c reductase complex, is a central component of the mitochondrial respiratory chain. The most frequent cause of CIII deficiencies is a mutation in the ubiquinol-cytochrome c reductase synthesis-like (BCS1L) gene, which was first identified by Petruzzella et al in 1998.^[1]^ The BCS1L gene is located on chromosome 2 (2q35) and encodes an ATPase that is associated with various cellular activities and is embedded in the mitochondrial inner membrane; this ATPase is presumed to facilitate the insertion of the Rieske Fe/S protein into precursors of CIII during the assembly of the respiratory chain.^[2]^

Herein, we report the first case of a compound heterozygous mutation in the BCS1L gene associated with CIII deficiency and Björnstad syndrome in a 7-month-old Chinese girl.

## 2 Case presentation

**Figure 1:**
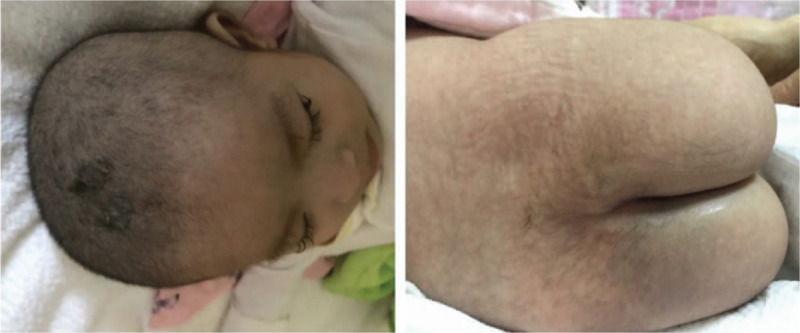
Presentation of sparse hair, twisted eyelashes and excessive hair growth on the back.

A 7-month-old girl presented to the Department of Pediatric Neurology with a 3-month history of psychomotor developmental retardation and a 1-month history of epilepsy combined with parallel psychomotor developmental deterioration. Her parents were nonconsanguineous, and her family history was unremarkable. The patient was normally delivered at full term, and her mother experienced no abnormality during pregnancy. The postnatal Apgar score and birth parameters were normal. The global developmental delay was considered based on disability in raising her head until 4 months old. She developed infantile spasms followed by parallel psychomotor developmental deterioration and presented with inability to raise her head at 6 months. The seizures manifested as repeated nodding with limb stretching (20-100 times daily). Physical examination showed a weight of 6.4 kg (3-10% percentile of the standard weight), a height of 63 cm (<3% percentile of the standard height), a head circumference of 40 cm (<3% percentile of the standard head circumference), sparse hair, twisted eyelashes, excessive hair growth on her back (Fig. 1), sucking weakness and reduced voluntary activities. A neurological examination showed decreased tension in the extremity muscles, normal tendon reflex, and positive bilateral pathological reflexes. The result of brain magnetic resonance imaging and brainstem auditory evoked potential were normal. Video electroencephalography revealed a series of spasm seizures and hypsarrhythmia in the bilateral occipitotemporal region. The Gesell Developmental Scales showed severe developmental delay in adaptive behavior, gross motor, fine motor, language and personal-social behavior. The results of gas chromatography-mass spectrometry were normal. Laboratory examinations revealed elevated transaminases (aspartate aminotransferase 336 U/L, normal range 13-35 U/L; alanine aminotransferase 503 U/L, normal range 7-40 U/L; glutamyl transpeptidase 103 U/L, normal range 7-45 U/L; alkaline phosphatase 314 U/L, normal range 35-100 U/L), elevated myocardial enzyme (creatine kinase isoenzyme 88 U/L, normal range<25 U/L), hyperammonemia (201 μmol/L, normal range 9-47 μmol/L), metabolic acidosis (pH 7.32, HCO_3_^−^ 14.8 mmol/L, BE -4.2 mmol/L, Lac 2.3 mmol/L) and positive urine glucose. The rest of the laboratory results, including urea nitrogen, creatinine, iron metabolism, fasting plasma glucose and thyroid function, were normal.

**Figure 2:**
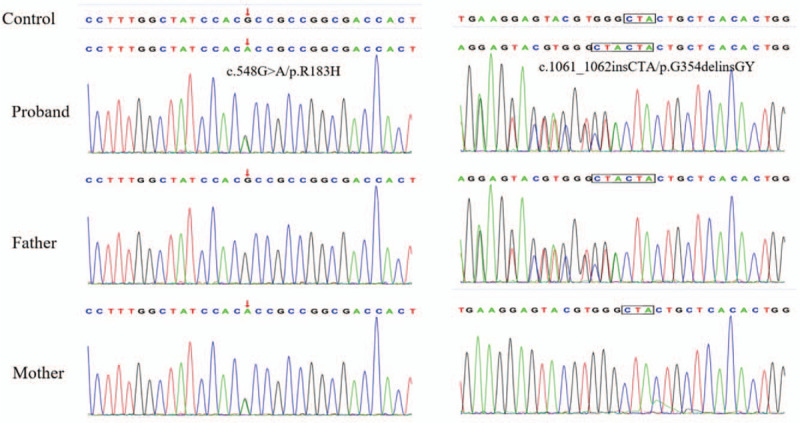
Sequencing of the BCS1L gene revealed a compound heterozygous mutation with a missense mutation (c.548G > A/p. R183H) inherited from the patient's mother and an insertion mutation (c.1061_1062insCTA/p. G354delinsGY) inherited from the patient's father.

Whole exome sequencing revealed a compound heterozygous mutation with a missense mutation (c.548G > A/p. R183H) in exon 4 of the BCS1L gene and an insertion mutation (c.1061_1062insCTA/p. G354delinsGY) in exon 8 of the BCS1L gene. Further genetic investigation showed that her mother harbored the former missense mutation, and her father harbored the latter insertion mutation (Fig. 2). Sequence analysis of the entire mitochondrial genome excluded a pathogenic mtDNA mutation.

Before admission, the patient had received oral topiramate (37.5 mg/d, 5.8 mg/kg/d) for 1 month, and the spasm seizures had not decreased. After admission, additional intravenous arginine hydrochloride (2.5 g/d) was administered for 5 days in the acute metabolic disorder phase, and persistent cocktail therapy was introduced, including coenzyme Q10 (20 mg/d), carnitine (1 g/d) and vitamins (vitamin B1, vitamin B2, vitamin B6, and vitamin C). The spasm seizures were decreased by 50% after 2 weeks of treatment. The blood ammonia, myocardial enzyme and urine glucose levels declined to normal levels. At a 1-month follow-up, the patient improved clinically with a decrease in spasm seizures of 75%, stronger sucking and more voluntary activities. However, she still had mild lactic acidosis (2.1 mmol/L) and mild hepatic damage (aspartate aminotransferase 98 U/L; alanine aminotransferase 143 U/L; glutamyl transpeptidase 99 U/L; and alkaline phosphatase 217 U/L).

The study was reviewed and approved by the local ethics review committee of the First Hospital of Jilin University. Informed written consent was obtained from the patient's guardians for publication of this case report and accompanying images.

## 3 Discussion

CIII catalyzes the electron transfer from succinate and nicotinamide adenine dinucleotide-linked dehydrogenases to cytochrome c. The most frequent cause of CIII deficiencies is a mutation in BCS1L, which was first identified by Petruzzella et al in 1998;^[1]^ to date, 38 different variants in BCS1L have been reported on ClinVar. BCS1L encodes a 419-amino acid-long protein and acts as a chaperone/translocase to facilitate the insertion of the Rieske Fe/S protein for the final step of CIII assembly in the inner mitochondrial membrane. The spectrum of clinical phenotypes that are caused by BCS1L gene variants ranges from severe growth restriction, aminoaciduria, cholestasis, iron overload, lactic acidosis and early death syndrome (growth restriction, aminoaciduria, cholestasis, iron overload, lactic acidosis and early death) to multisystemic CIII deficiency, which is characterized by encephalopathy of variable severity, proximal tubulopathy and liver failure and mild Björnstad syndrome with sensorineural hearing loss and a brittle hair condition known as pili torti.

We report the first case of pathogenic BCS1L variants associated with CIII deficiency and Björnstad syndrome in China. The patient carried 2 distinct heterozygous mutations in BCS1L. The R183H variant, which was inherited from her mother, had been previously reported to be associated with the pathogenesis of Björnstad syndrome and had been recorded in OMIM, HGMD and ClinVar databases.^[3]^ The G354delinsGY variant, which was inherited from her father, was novel and had not been listed in above genetic databases. The phenotype of this patient appeared to have hallmarks of CIII deficiency associated with defects in BCS1L and severe encephalopathy (psychomotor developmental retardation and infantile spasms), tubulopathy (positive urine glucose), live failure (elevated transaminases and hyperammonemia) and lactic acidosis. It is remarkable that the patient had pili torti but no sensorineural hearing loss according to the normal brainstem auditory evoked potential. As a result, the presented phenotype is between Björnstad syndrome and CIII deficiency. The clinical phenotype largely depends on the genotype. The phenotype of compound heterozygous mutations could be determined by considering the phenotypes of patients who are homozygous for mutations near 1 of these 2 mutation sites. The R183H mutation has been previously observed to be associated with Björnstad syndrome. Although the G354delinsGY mutation has not been previously reported, it may be near V353M, which has been reported and associated with CIII deficiency.^[4]^ We hypothesize that the phenotype of this novel mutation is CIII deficiency, but it requires more published cases or gene function identification. Although it remains to be determined why BCS1L variants exert such a varying spectrum of clinical phenotypes, patterns in specific amino acid substitutions and positioning within the BCS1L structure,^[5]^ tissue-specific manifestation of BCS1L may provide additional clues regarding how to improve phenotype-genotype correlations.^[6]^

To the best of our knowledge, seizures have been reported in 7 cases, and the types of seizures included focal seizures and drug-resistant epileptic spasms. Additionally, specific treatment options for this disease remain a challenge. Our patient presented with severe spastic seizures. Although oral topiramate, as an antiepileptic drug, was not effective, we did not prescribe additional ACTH therapy or other antiepileptic drugs partially because of live injury and metabolic disorders. We chose dietary supplements due to her presentation, including growth and psychomotor developmental retardation, hypertrichosis, hepatic and renal pathologies, metabolic disorders, and normal gas chromatography-mass spectrometry. Significant improvements in the seizures and psychomotor development were achieved. However, the patient still had mild lactic acidosis and mild hepatic damage. Recent research in mouse models suggests that a ketogenic diet may improve the hepatic symptoms that often accompany BCS1L mitopathies.^[7]^ We recommend that a ketogenic diet should be initiated to control refractory epilepsy and to improve hepatic damage. However, the patient's parents refused.

We reported the first patient with CIII deficiency and Björnstad syndrome in China and identified 1 novel mutation (C.1061_1062insCTA and P. G354delinsGY) in the BCS1L gene. This finding expands the BCS1L gene mutation profile and will be beneficial for genetic diagnosis.

## Author contributions

**Conceptualization:** Xuncan Liu, Jianmin Liang.

**Data curation:** Xuncan Liu, Chen Chen.

**Investigation:** Yanfeng Zhang, Jianmin Liang, Si Yang, Chen Chen.

**Methodology:** Xuncan Liu, Yanfeng Zhang, Jianmin Liang, Si Yang, Chen Chen.

**Supervision:** Xuncan Liu, Yanfeng Zhang, Jianmin Liang, Si Yang.

**Writing – original draft:** Xuncan Liu, Yanfeng Zhang, Chen Chen.

**Writing – review & editing:** Xuncan Liu, Chen Chen.
